# How Immunocontraception Can Contribute to Elephant Management in Small, Enclosed Reserves: Munyawana Population as a Case Study

**DOI:** 10.1371/journal.pone.0027952

**Published:** 2011-12-09

**Authors:** Heleen C. Druce, Robin L. Mackey, Rob Slotow

**Affiliations:** Amarula Elephant Research Programme, School of Biological and Conservation Sciences, Westville Campus, University of KwaZulu-Natal, Durban, South Africa; Australian Wildlife Conservancy, Australia

## Abstract

Immunocontraception has been widely used as a management tool to reduce population growth in captive as well as wild populations of various fauna. We model the use of an individual-based rotational immunocontraception plan on a wild elephant, *Loxodonta africana*, population and quantify the social and reproductive advantages of this method of implementation using adaptive management. The use of immunocontraception on an individual, rotational basis stretches the inter-calving interval for each individual female elephant to a management-determined interval, preventing exposing females to unlimited long-term immunocontraception use (which may have as yet undocumented negative effects). Such rotational immunocontraception can effectively lower population growth rates, age the population, and alter the age structure. Furthermore, such structured intervention can simulate natural process such as predation or episodic catastrophic events (e.g., drought), which regulates calf recruitment within an abnormally structured population. A rotational immunocontraception plan is a feasible and useful elephant population management tool, especially in a small, enclosed conservation area. Such approaches should be considered for other long-lived, social species in enclosed areas where the long-term consequences of consistent contraception may be unknown.

## Introduction

Within natural, open conservation systems, large stochastic events such as drought, fire and predation keep populations at a sustainable level by eliminating the old, weak and young [1], [2], [3], [4]. However, within modern conservation areas, especially small, enclosed reserves, natural stochastic events are altered by human management interventions [3], with more intervention required for smaller reserves [5]. Within these conservation areas, the occurrences and spread of big fires are often prevented or controlled [4], while natural droughts have limited effects on wildlife populations, as critical resources are usually never a limiting factor due to water and food provision [6], [7]. The fences prevent natural movement patterns from and into these areas, and predation events are effected and controlled within these areas, as managers determine and restrict the predator-prey ratios, and predator population structure [5], [8]. This can result in eruption of populations which leads to significant environmental problems [9], [10], which then require active management intervention [11], [12], [13], [14].

Natural processes should be simulated to achieve management objectives without a negative effect on the system [15]. However, because active management requires managers to impede the natural processes of nature [16], it can often have unforeseen consequences (e.g. killing of rhino, *Ceratotherium simum*, by elephant, *Loxodonta africana*) [17]. This is of special concern for species with complex social systems, e.g. Hamadryas baboons, *Papio hamadryas* [18], [19], Lion-tailed Macaques, *Macaca silenus* [20] and elephants [17], [21]. Thus, for management interventions to be effective and non-detrimental, a sound understanding of the natural processes is required.

Small, enclosed reserves within South Africa are experiencing eruptive elephant population growth, which is an increasing concern to conservation biologists, ecologists and wildlife managers [22], [23], [9]. In the older, larger populations, these elephants were introduced as orphans from culls in Kruger National Park [23]. These introductions have resulted in very young, fast-growing populations, with no or very low, adult senescence [11], [23]. The pressure exerted by increasing density of animals can cause environmental damage [10] and changes in biodiversity [24], [25], [26]. Therefore, overabundance and rapid growth rates may require active management [27], [28].

There are two natural processes that could control elephant population numbers. One process is natural mortality, particularly of young animals [3], [4]. During episodic catastrophic events (e.g., drought), entire cohorts of juvenile elephants can be lost [2], [3]. The second process is the regulation of female inter-calving intervals (and, less importantly, age of maturation - [11]) by environmental conditions [29]; under adverse conditions, inter-calving intervals should increase [30].

Immunocontraception has been used as a management tool around the world for numerous years to restrict rapid population growth in captive as well as wild populations of many animal species i.e. feral horses (*Equus caballus*) [31], [32], [33]; Prezewalski's horses (*Equus prezwalskii*) and banteng (*Bos javanicus*) [34]; white-tailed deer (*Odocoileus virginianus*) [35], [36]; Brandt's vole (*Microtus brandti*) [37]; Tule elk (Cervus *elaphus nanodes*) [38]; and African elephants [39], [40], [41]. Immunocontraception of African elephants has proven safe [41], [42] and effective in reducing population growth rates [41], [43], [44], [45]. Consequently, immunocontraception can be used to prevent female elephants from conceiving, or to increase the span of calving intervals of each individual female, and thereby reduce population growth. However, immunocontraception can reduce the existing population size only when it decreases the birth rate to a level that is below the mortality rate. This reduction in birth rate will subsequently age a population over the long term [46], assuming that age-specific mortality rates are constant. By preventing calving or by prolonging calving intervals, immunocontraception can be used to simulate calf mortalities from predation or prolonged bouts of adverse environmental conditions (e.g. droughts).

Immunocontraception has a minimal influence on elephant social behaviour in the medium term [41], [42], [44]. However, it has been suggested that social problems may occur in elephant populations treated with prolonged use of immunocontraception that is intended to prevent any calves being born into a population [47], [48]. Potential social problems include the lack of allomothering experience within family groups, due to prolonged absence of newborn calves, and depression amongst adult females arising from their continual oestrus cycling as an inability to conceive and give birth [48]. To overcome these potential long-term effects, females can be allowed to give birth periodically. The effects of such births on populations, and how to manage such reversal of contraception at a population level, is unknown. The rotational use of contraception can simulate natural processes within a small, enclosed population, but it remains important to monitor and study the social and behavioural effects.

This study attempted to reveal some knowledge and understanding on the rotational contraception on a species at the population level. The feasibility of implementing individual-based contraception of elephants has been demonstrated elsewhere [49]. Here we used the Munyawana elephant population as a case study to demonstrate an example of individually-based, rotational immunocontraception used to simulate the effects of natural mortality which increase inter-calving intervals. We use population models to determine potential effects of immunocontraception-based management plans on elephant population size and age structure.

## Methods

### Study Area

**Figure 1:**
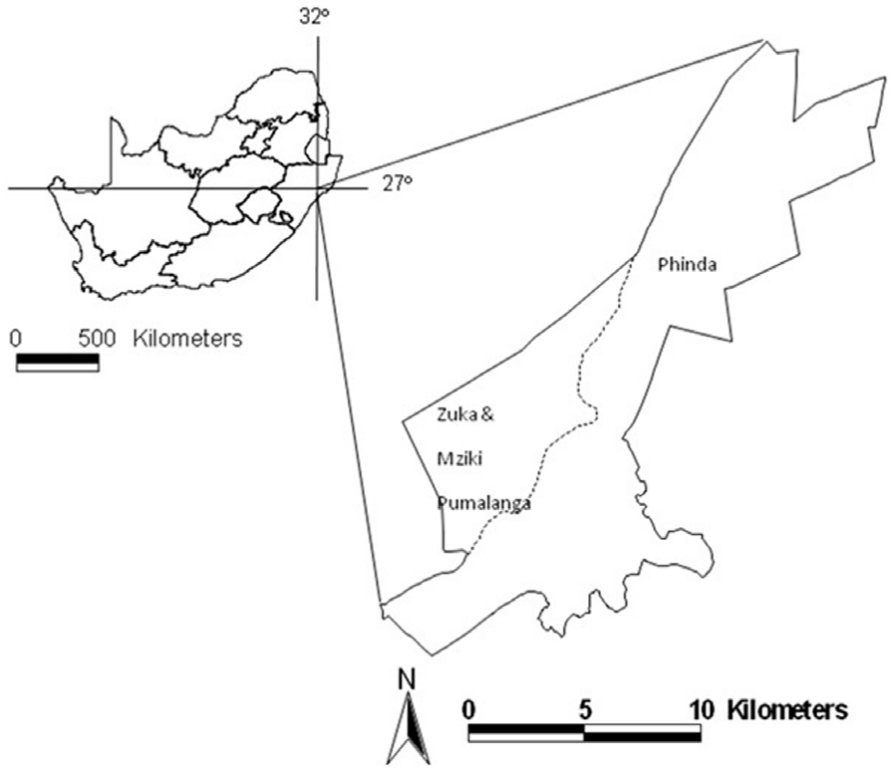
Munyawana Conservancy. The dashed line indicates the position of the boundary fence between Phinda and the new sections of Zuka, and Mziki Pumalanga before the fences were removed during August 2004.

This study was conducted within the Munyawana Conservancy, KwaZulu-Natal, South Africa (27°51′30″S, 32°19′00″E). Initially, Phinda Private Game Reserve (Phinda) was established in 1991, with an area of approximately 150 km^2^. During August 2004, the boundary fences between Phinda and two neighbouring reserves, Zuka and Mziki Pumalanga were removed, forming the Munyawana Conservancy (185 km^2^) (see Fig. 1). During May 2006, the boundary fences were removed between Munyawana Conservancy and the neighbouring reserve, Sutton, increasing the area of the conservancy to 207 km^2^ [7].

The vegetation types within the Munyawana Conservancy were Sand Forest ([50]; Type 3), Sweet Lowveld Bushveld ([50]; Type 20), Natal Lowveld Bushveld ([50]; Type 26), Lebombo Arid Mountain Bushveld ([50]; Type 13) and Coastal Bushveld-Grassland ([50]; Type 23). One perennial river, the Mzinene River, flows from west to east through the southern section of the conservancy, and dams were extensively distributed throughout the properties. During the rainy season, surface water was extensive; while some of these dams retain water all year round, other dams were supplied with borehole water during the dry periods, i.e. water was always available. The Munyawana Conservancy has a summer rainfall regime and temperatures range from an annual mean minimum of 10°C to an annual mean maximum of 35°C.

### Munyawana Immunocontraception Management Plan

The Munyawana management team was greatly concerned about the continuous elephant population growth within the small and enclosed system. By the end of 1994, a total of 58 elephants had been introduced into Phinda from Gonarezhoa in Zimbabwe and from former Kruger culling operations [51]. Within 10 years, the Phinda elephant population almost doubled in numbers, with the average annual population growth rate since introduction equalling 9.4%. The elephant population was monitored on a daily basis from March 2003 through to July 2006 (end of data used in this study, but monitoring is still continuing in 2009). As many elephant as possible were located each day, and general location data, identities of adult individuals present and behavioural activities (in general, as well as musth, oestrus behaviours and newborn calves) were recorded. All population demographic data until July 2006 were used in the models. Monitoring of the populations within the inclusive reserve began once these areas became part of the conservancy. All individual elephants were known, as well as the family groupings.

During July 2003 the population was reduced from an estimated 107 individuals to 66 individuals through the translocation of four family groups to other private game reserves in South Africa. In July 2006, the total elephant population within the Munyawana Conservancy consisted of 98 individuals, with 20 independent bulls and seven family groups. Of this, the Phinda population comprised 88 individuals, with 19 independent bulls and five family groups. The Zuka population consisted of three young individuals and the Sutton elephant population comprised one family unit made up of seven individuals. Neither the Zuka nor the Sutton populations amalgamated into the Phinda population during this study period, and the Sutton group has subsequently (during November 2007) been translocated from the reserve.

The 2003 translocations reduced the breeding population to a more manageable size (21 sexually mature females) and during May 2004 an immunocontraception plan (ICP) was implemented. The aim of this ICP was to reduce the overall population growth rate, but not to completely prevent conception within the entire female population. The proposed ICP allowed young mothers to have their first calf before being included in the ICP. It also allowed females to calve on a rotational basis within each family group. Through this, the ICP aimed to increase the inter-calving interval of individual females within each family group, but to still allow the social needs of the family groups to be met, in that calves would still be born into the groups on a continuous and regular basis. Births would also be rotated between the females within each family group. The ICP allowed one young calf to be born into each family group at least every two to three years. A further aim of this ICP was to create a more natural population structure, with newborn births evenly spread over time. Herds derived from orphan populations tend to be synchronised in their calving as the introduced female orphans all tend to reach sexual maturity at the same time and, therefore, give birth to their calves at similar times ([23], H.C. Druce, pers. obs.). During the elephant immunocontraception darting operations, the contraceptive was administrated by methods described in detail [49]. All the immunocontraception darting procedures during 2004–2007 were done from ground, either from vehicle or on foot. Annually the same marksman administered the contraceptive remotely by means of drop-out darts fired from a Dan-Inject dart gun and thereafter darts were retrieved to ensure appropriate treatment.

### Immunocontraception Model

An individual-based rotational spreadsheet model was developed to make projections of the size, growth rate and age structure of the Munyawana elephant population under a set of potential management immunocontraception intervention plans. More specifically, we examined the effect of altered inter-calving intervals versus preventing females from conceiving their first calf upon sexual maturity. To determine the robustness of our projections, we tested the sensitivity of the model projections to realistic variations in the demographic parameters (age at sexual maturity, time to conceive after release from contraception, natural calving interval).

The demographic parameters incorporated in this model were: (1) age of sexual maturity of females (age of first oestrus, with assumption of first conception), (2) calving interval (average interval between consecutive births for a mother), (3) birth sex ratio, (4) maximum age of individuals, and (5) age at menopause (see [11] for parameter details and calculated methods). Additional management parameters modelled were: (5) contraception implementation age (allowing or preventing females from conceiving their first calf upon the age of sexual maturity), and (6) conception time (the time for a cow to conceive upon being released from contraception).

The parameter values were constant for the birth sex ratio, which was 1∶1 [11], [52], [53], [54], maximum age of individuals (60 years [11], [53], [54]) and the age of menopause (50 years [53], [55]). Female elephants may reach sexual maturity as late as 17 years [52], and will typically produce the first calf two years later [55], [56], [57]. However, Mackey [11], [58] calculated the average age of female sexual maturity in four small, enclosed reserves to be between 8 and 10 years. The average age of sexual maturity of the Munyawana population was previously thought to be 10 years [11], but additional data up to 2009 indicate this to be nine years. The inter-calving interval of cows is between four and five years [55], [59], [60], with estimates as high as four to nine years [61]. However, recent studies in enclosed populations in South Africa determined calving intervals at between three and four years [11], [58]. Again, newer census data up to 2009 (but before immunocontraception took effect) for Munyawana indicate average calving interval has reduced from four years [11] to three years.

Moss [59] observed that female elephants experience very short oestrus cycles of on average four days with females coming into oestrus throughout the year. Sufficient field testing has not yet been done, but estimates of the time for an elephant cow to conceive upon being released from contraception vary from 12 months [43], 12 to 18 months (D. Grobler, CatchCo Africa, pers. comm.), or may be approximately equal to the number of years an elephant cow has been subjected to vaccination [46].

**Figure 2:**
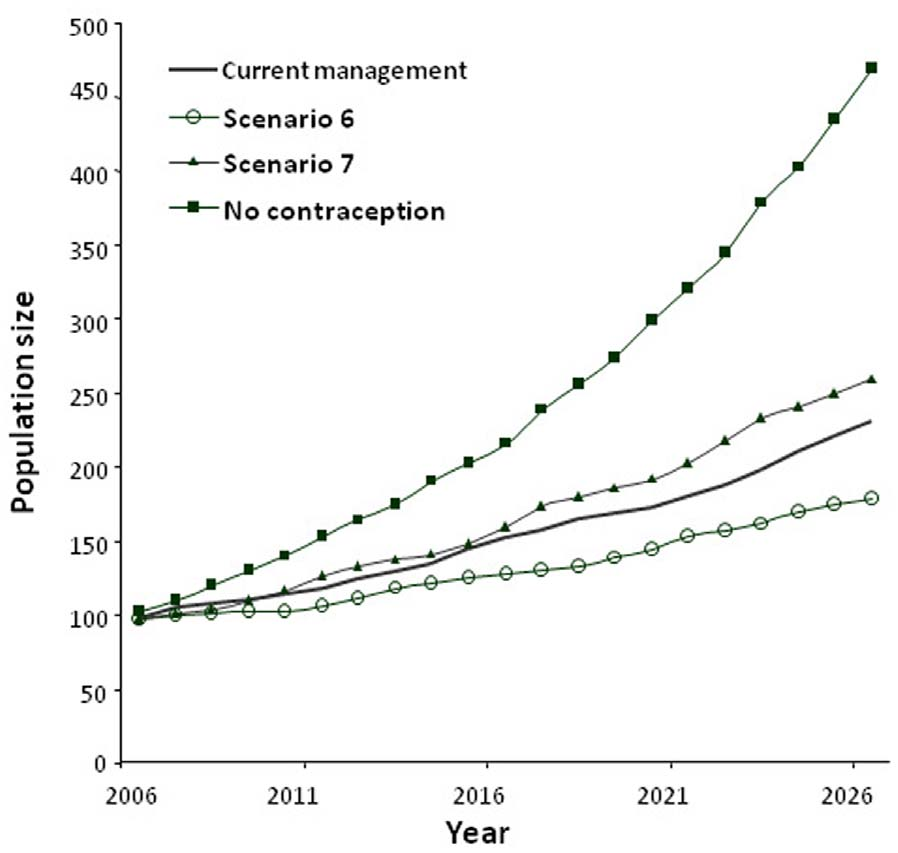
Projected population size for the Munyawana elephant population under different immunocontraception scenarios for a 20-year time period. Results are shown for the current Munyawana immunocontraception plan, no application of immunocontraception on the population, and two contraception scenarios (Scenarios 6 and 7) that resulted in the most extreme projections. Scenario 6 was the prevention of the first calf and allowing the female to calf at 19 years of age, with a baseline contraception-induced calving interval of 8 years thereafter. Scenario 7 examined a shortened calving interval of 6 years.

The different contraception scenarios were simulated by adjusting a single parameter per scenario and keeping the rest of the parameters at the baseline value (Table 1). We assumed contraception was 100% effective in preventing conception in treated females [41], [42]. Model simulations were done for 20 years (2006 to 2026) to obtain population projections on a timescale which is of relevance to management decision making (Fig. 2). Density dependent regulation was excluded from this simulation model because of the time-scales of the model, time-lags associated with the long generation times and 22-month gestation periods, and the young age structure of the population make changes in natural rates of senescence unlikely (As a young orphan introduced population, none of the adult elephants exceed the age of 60 within the 20 year modelled time frame). Similarly, no stochastic mortalities (drought, fire and predation) were included in the model, as the model was specifically aimed at the known Munyawana population and because, due to intensive macro-management within the small, enclosed environment, stochastic events are unlikely to impact the elephant population (artificial water sources are provided [62], fire is managed (pers. Obs.), and lion groups size kept small resulting in no lion predation of elephant [63]). The purpose of the model is to show the ability to manipulate the population, through selective interventions, to make it more natural in structure. Therefore to use individual-based rotational immunocontraception as an adaptive management tool to simulate natural mortality of young, along with natural environmental effects on female reproduction by ensuring some prolonged inter-calving intervals.

**Table 1:** Modelled elephant population growth rate, population doubling time and population size for the contraception period 2006–2026.

Modelled scenarios for Munyawana elephant population	Parameters				Annual growth rate (%)^v^	Population doubling time (years)^vi^	Projected population size	
	Age of sexual maturity (years)^i^	Implemen-ation age (years)^ii^	Concep-tion duration (years)iii	Calving interval (years)^iv^			2006 (start)	2026 (end)
Munyawana- current contraception plan	The combined Phinda & Sutton contacepted, Zuka non-treated plans				4.20	18	98	230
Munyawana- no-contraception plan	9	-	-	3	7.58	10	102vii	469
Scenario 1	9	10	1	8	4.16	18	98	217
Scenario 2	9	10	3	8	4.13	18	98	216
Scenario 3	8	9	2	8	4.36	17	98	228
Scenario 4	9	10	2	8	4.15	18	98	216
Scenario 5	10	11	2	8	4.03	19	98	211
Scenario 6	9	8	2	8	3.19	>20	98	178
Scenario 7	9	10	2	6	5.06	15	98	259
Scenario 8	9	10	2	10	3.48	20	98	196
**Current contraception plan for the individual elephant populations within the Munyawana Conservancy**								
Phinda	9	11	3	8	3.71	19	87	184
Zuka-no-contraception	9	-	-	3	8.73	6	4	25
Sutton	8	10	3	9	5.26	12	7	21

i Parameters for the age of sexual maturity were 8 years, 9 years (baseline) and 10 years.

ii Parameters for the contraception implementation age were 8 years (prevent the first calf and only allow the first calf at 19 years after allowing an 8 year calving interval) or 10 years (baseline – allows the first natural birth, if the cow conceive at the baseline of 9 years age at sexual maturity).

iii The length of time that a female was released from contraception to ensure conception, with the parameters of 1 year, 2 years (baseline) and 3 years.

iv Parameters for the contraception induced calving intervals were 6 years, 8 years (baseline) and 10 years.

v The growth rate was calculated for the 20-year time span (2006–2026) from the slope of regression on the natural log of population size against year.

vi The time it takes for the population to double the starting numbers.

vii The Munyawana elephant population total at the beginning of 2006, was calculated as if no females were on contraception for the past 3 years and would have conceived, accordingly a calving interval of 3 years was maintained from the age of the youngest calf.

The parameters for the eight individual modelled scenarios and the current implemented immunocontraception plans within the Munyawana population are presented within the table above.

**Figure 3:**
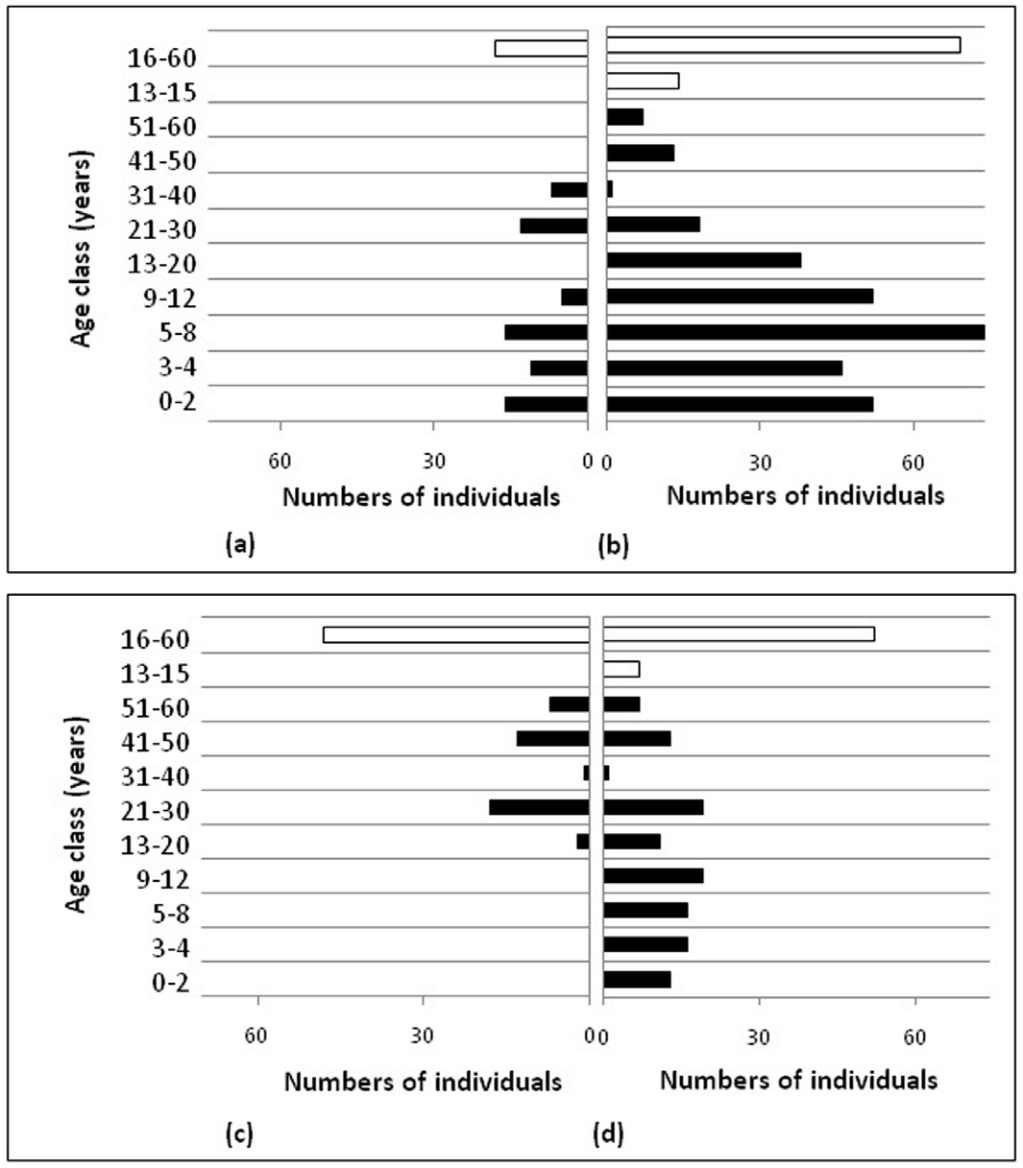
The projected Phinda elephant population divided into age classes represented as absolute numbers under different immunocontraception scenarios. The age classes are classified as 0–2 years: infant, 2–4 years: juvenile, 5–8 years: intermediate, 9–12 years: sub-adults, older than 13 years are classified as an adult. Adult bulls are presented by white bars (with only two age classes), while all the individuals in the breeding herds are represented by the black bars which include males <13 years. (a). The Phinda elephant population in 2006 before any effects of immunocontraception had taken affect. The projected Phinda elephant population in 2026, (b). without any application of immunocontraception. (c). with a 100% application of immunocontraception. (d). with a rotational application of immunocontraception, as the current Phinda implemented immunocontraception plan.

**Figure 4:**
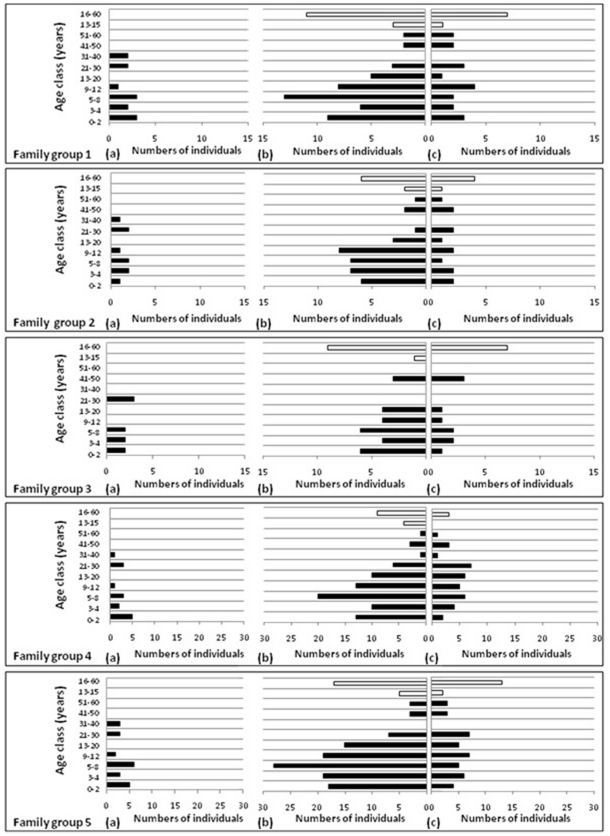
The projected Phinda elephant population as family groups and divided into age classes represented as absolute numbers under different immunocontraception scenarios. The age classes are classified as 0–2 years: infant, 2–4 years: juvenile, 5–8 years: intermediate, 9–12 years: sub-adults, older than 13 years are classified as an adult. Independent adult bulls born into family groups are presented by white bars (with only two age classes), while all the individuals in the family groups are represented by the black bars which include males <13 years. (a). The Phinda elephant population in 2006 before any effects of immunocontraception had taken affect. The projected Phinda elephant population in 2026, (b). without any application of immunocontraception. (c). with a rotational application of immunocontraception, as the current Phinda implemented immunocontraception plan.

The age structure of the population was determined by assigning each individual into one of five age classes (infant, juvenile, intermediate, sub-adult and adult). The adult age class was further sub-divided into smaller age categories (see breakdown in legends of Fig. 3 and Fig. 4). The absolute numbers of individuals per each age class were calculated at the end of the final year of the simulation (i.e. 2026). The age structure was calculated for the entire population as well as each family group/herd.

## Results

Changes in projected population size and growth rate were described for a 20-year span (2006–2026) of the actual contraception plan (as decided by the Munyawana management team separately for the three populations –Phinda, Zuka and Sutton), other contraception scenarios and no-contraception application. The projected effects of contraception on elephant population size showed that there was a large difference in population size over a 20-year period between a non-treated population and a treated population (Table 1, Fig. 2). Annual growth rates for the 20-year period for a non–treated population was 7.58% versus 4.2% for the Munyawana immunocontraception plan that is currently being implemented (Table 1). The slowest overall growth rate was 3.19% for the Munyawana population (Scenario 6) in which females were prevented from conceiving their first calf until 8 years after achieving sexual maturity – producing the first calf at 19 years. The highest projected value (5.06% annual growth rate) for any scenario with contraception was Scenario 7, which had a 6-year calving interval (Fig. 2).

Under the current immunocontraception plan, the Munyawana population would double after 18 years, while the same population would double within 10 years without any contraception implementation (Table 1). When the calving interval was lengthened to longer than 6 years and prevention of the first calf (such as Scenario 6 and 8) was implemented, the population doubling time was projected to be 20 years or longer.

The Zuka population, which is not under a contraception program, had the greatest overall growth rate of 8.73%. If the Zuka population continues to be left out of the contraception plan, it will double in only 6 years.

Sensitivity analyses indicate the response of the projected elephant population growth rates to changes in the demographic parameters of the model, or the robustness of model projections to change in demographic parameters. Population projections were most sensitive to changes in calving interval and the implementation age of contraception (i.e. whether a female's first calf was delayed). Changes in calving interval produced relatively large changes in population growth rate, with an increase from six to ten years resulting in a reduction of 1.58% in annual growth rate (calculated over 20 years) from 5.06% to 3.48%. Changes in implementation age of contraception from ten to eight years (i.e. if sexual maturity is at nine years of age, therefore by delaying the first born calves), produced a reduction of 0.95% in annual growth rate. The model projections were not particularly sensitive to age of sexual maturity and the length of conception time after release from contraception. Changes in age of sexual maturity produced relatively small changes in population growth rate, with an increase from eight to ten years resulting in a reduction of 0.33% in annual growth rate (from 4.36% to 4.03%). Increasing the conception time from one to three years resulted in a reduction of only 0.03% in annual growth rate (from 4.16% to 4.13%).

The model was used to project the probable changes to the age structure of the population under various contraception scenarios (Fig. 3). The initial population age structure before any immunocontraception had taken affect during 2006 was used as the baseline data (Fig. 3a) to simulate different future outcomes, where after comparisons of age structure were made between no-contraception, 100% and a rotational contraception from predicted model results at year 20 (i.e. 2026). When no-contraception was applied to the Munyawana population, the model projections indicated that the bulk of the population comprised young animals, and as the breeding population increased in size over time the recruitment of young also increased (Fig. 3b). When a continual 100% contraception rate was applied, there were no new calves added to the population and the average age of individuals in the population has increased; this ultimately had the effect of aging the population (Fig. 3c). With rotational immunocontraception application, the Munyawana population produced a limited number of calves, subsequently resulting in a more even age structure (Fig. 3d). The population age structures for 100% immunocontraception were very different from those projected for rotational contraception scenarios.

The total number of adult females (females older than 13 years of age) at the end of 2026 for the 100% contraception rate was 41 (Fig. 3c), with 77 adult females for the no-contraception application (Fig. 3b) and a total of 51 adult females for the rotational contraception application scenario (Fig. 3d). The number of adult females present within the population indicates the reproductive potential and future growth rate.

Similar projected effects were found on the age structure of individual family groups/herds and that of the overall Munyawana population under the various contraception scenarios (Fig. 4a, 4b, 4c). At the end of the 20-year modelled period under rotational contraception, the average age of individuals in the family group had increased and their growth rates had been reduced, but they still contained calves that had been born into each group over the period (Fig. 4c). A large number of independent males were contained in family group 3 as a result of a male-biased calving documented in this family group during 2006, whereas family groups 4 revealed a female calf-biased during 2006 which results in a larger amount of reproductive females at the end of the 20-year modelled period.

## Discussion

Immunocontraception is a tool that can be adapted to meet different management objectives in reducing population growth [41], [49], [64]. This study showed that a rotational approach to an immunocontraception plan can be a useful tool to age a population and thereby stabilise its age structure; yield a reduced population growth and prevent irruption of young populations; allow for management of populations, family groups and individuals in relatively small reserves enclosed by fences.

The current Munyawana immunocontraception management plan approximately halved the population growth rate and doubled the population's doubling time, compared to when no-contraception was implemented. The results also provide some insight into which demographic parameters may be most important for determining rate of population growth. Mackey [11] also concluded that calving interval was more important for regulating elephant population growth than any other parameters we evaluated.

The sensitivity analyses indicated little change in population growth from variation in the other parameters, showing that the model is fairly robust. The magnitude of natural variation in demographic parameters should have little effect on model projections. Due to this projected relative insensitivity of elephant population growth to variation in demographic parameters, extremely complex immunocontraception plans may not necessarily be required. What will have the greatest effect on population growth is whether the population is treated or not; potential natural variation in demographic parameters in the short- and medium-term will lead to only minor effects on population growth. However, the population age and sex structure, as a demographic parameters are important to determine future reproductive potential, especially if management ceases future contraception treatment. The age structure will be affected by the natural old age senescence within a population and the proportion of births will be directly related to the proportion of adult females in the population at the time.

With a rotational immunocontraception plan, the population should undergo a stabilisation of the age structure. This should result when annual recruitment is reduced to the same level as senescence (the only significant source of elephant mortality in South Africa's small enclosed reserves, but see [4]). Alternatively, for a more extreme effect, a contraception rate of 100% over a long term would result in no calves being added to the population with the consequence that the population would age, due to the average age of individuals in the population increasing over time. If this rate were applied over a longer time period, it would result in a decrease in the population through senescence without births, a possible alternative to culling.

The long-term effects of immunocontraception of female reproductive health are still uncertain. Delsink [45] showed that ovulation and oestrus cycles remained the same after five years of continuous immunocontraception of female elephants. Immunocontraception is said to be reversible by some researchers [42], [43], [44], but some studies have shown that the continuous long-term use of the immunocontraception vaccine porcine zona pellucida (PZP) may cause ovarian disfunctioning [34], a slow return of fertility [65] or even the permanent loss of fertility [34]. The possibility that the long-term use of PZP might cause infertility in elephant females still needs to be tested [47], [64].

However, many of the social and behavioural concerns previously raised about prolonged, continuous and indefinite use of immunocontraception in elephants may be reduced, or eliminated, by the use of a rotational, individual-based contraception program. Concerns have been raised about the negative effects on group behaviour that could arise from immunocontraception plans that completely prevent offspring being born into a herd [40], [66]. Additional negative effects may include changes in feeding patterns and spatial use [48], the lack of allomothering (as described by [67]) affecting the learning of first-time mothers [48], and depression in mature females resulting from their inability to calve for a long period of time [48]. Because a rotational, individual based immunocontraception plan would permit all females to calve, but with prolonged inter-calving intervals, these potential negative effects of contraception should be reduced. Thus, immunocontraception following such a plan should not pose significant social or behavioural concerns and/or threats.

Managers of large reserves with a high elephant population density may question the realistic effect of immunocontraception as a management tool. Delsink [41] suggested a ‘mass-darting approach’ for large populations, which is a more flexible approach than the individual-based approach. When a large population of elephants is known on a herd/family group level, the rotational mass darting approach could be applied to family groups/herds within a population, whereby contraception darting can be rotated between herds at a management determined time period. The better the knowledge of an entire elephant population's demographics, the more feasible immunocontraception becomes. Further modelling and future work on testing mass application methods will need to be undertaken.

Stochastic events naturally control the population growth rate, size and age structure, while eliminating the population's old, sickly, weak and young [1], [2], [3]. Where management either controls or prevents the occurrence of normal natural stochastic events, eruptive populations arise, especially within small, enclosed conservation areas [11]. The simulation of natural events (like drought and predation) by management will have consequences on the population demographics and behaviour, which might result in problem behavioural responses as seen in elephants [17], [68], [69], predators [70] and primates [18], [19]. Therefore management requires a sound understanding of the natural processes, social demographics and behavioural requirements applied to the specific species involved. Hereafter with this understanding and essential monitoring, simulation of natural processes can be used in adaptive management plans.

### Management Implications

Immunocontraception can be used as a tool to simulate natural stochastic events like drought, however a continual drought with complete calf mortality (e.g. by implementing a 100% contraception continually) is not natural. Therefore rotational immunocontraception can be used to simulate drought cycles, whereby four years of drought are simulated and thereafter four years of non-drought, which would allow cohorts of births to occur. Another approach can be to simulate predation events by using an individual-rotational immunocontraception application approach, whereby selected females are treated and prevented from conceiving, as to simulate that those calves are removed from the population. Therefore rotational, individual-based immunocontraception can be a useful, practical, effective and flexible management tool to include as part of an adaptive elephant management plan.

### Ethical Approval

Ethical approval for the use of the vaccine was obtained from University of Pretoria's Animal Care and Use Committee, Project number: 36-5-251 (Project name *Non-lethal control of African elephant (Loxodonta africana): Game reserves and respective elephant populations*).

The South African Medicines Control Council issued permits and approval for the “Use of an unregistered medicine in terms of Section 21 of Act 101 of 1965”. (Permit numbers SP/35/2002, SP/11/2003, SP/51/2004 and SP/166/2004) [71]. During the elephant immunocontraception darting operations, the contraceptive was administrated by approved methods, as described in [48], [45].
